# Prevalence and Risk Factors of QTc Prolongation During Pregnancy

**DOI:** 10.3389/fcvm.2021.819901

**Published:** 2022-01-24

**Authors:** Chaodi Luo, Zhenzhen Duan, Yi Jiang, Peng Liu, Yang Yan, Dan Han

**Affiliations:** ^1^Department of Cardiology, The First Affiliated Hospital of Xi'an Jiaotong University, Xi'an, China; ^2^Department of Cardiovascular Surgery, The First Affiliated Hospital of Xi'an Jiaotong University, Xi'an, China

**Keywords:** QTc prolongation, twin pregnancy, prevalence, risk factors, single pregnancy

## Abstract

**Background:**

Prolonged QT intervals have been observed in pregnant women, which predispose them to a higher risk of potentially lethal ventricular arrhythmias. This study was designed to evaluate the prevalence of QTc prolongation in Chinese hospitalized parturient women with single and twin pregnancies, and to explore potential risk factors associated with QTc prolongation.

**Methods:**

This retrospective study included 1,218 patients from a large Chinese population between January 2014 and October 2020. Data from parturient women with single and twin pregnancies without pre-pregnancy cardiac diseases were collected. QTc was corrected by the Fridericia formula [QTc = QT/RR(1/3)], and QTc ≥ 460 ms for females was defined as prolonged QTc, QTc ≥ 500 ms was defined as severely prolonged QTc. The prevalence and common risk factors of QTc prolongation during pregnancy were analyzed in this cohort. Uni- and multivariable logistic regression analysis were performed to identify clinical parameters associated with QTc prolongation in this population.

**Results:**

The prevalence of QTc prolongation was 48.19% among this population, 10.56% in single pregnancy, 89.44% in twin pregnancies. The prevalence of severely prolonged QTc was 23.48% among the total cohort, 0.49% in single pregnancy, and 46.47% in twin pregnancies. The mean QTc interval was significantly longer in twin pregnancies than in single pregnancy (498.65 ± 38.24 vs. 424.96 ± 27.67 ms, *P* < 0.001). Systolic blood pressure, diastolic blood pressure, total cholesterol, serum uric acid, gestational hypertension and twin pregnancies were associated with QTc prolongation in parturient women.

**Conclusion:**

This is the first study to assess the prevalence and risk factors of QTc prolongation between single and twin pregnancies. QTc prolongation is more prevalent, and QTc intervals are significantly longer in twin pregnancies as compared to single pregnancy.

## Introduction

The heart accommodates numerous anatomical and physiological changes to support the increased metabolic demands of maternal organs and fetal development during pregnancy. These changes result in cardiac electrical remodeling that can often be detected as alterations in the electrocardiogram (ECG) such as an increased heart rate and lengthening of the QT interval (1). Physiological changes and obstetric complications significantly enhance the susceptibility of arrhythmic activity during pregnancy and the postpartum period, especially in the context of multiple pregnancy (2). Data showed that the prevalence of cardiac arrhythmias in the general pregnant population is 166/100,000, among which supra-ventricular arrhythmia (SVT) is 24/100,000 and ventricular arrhythmia (VT) is 2/100,000 (3). Pregnancy-related death is defined as the death of a woman during or within 1 year of pregnancy that was caused by a pregnancy complication, a chain of events initiated by pregnancy, or the aggravation of an unrelated condition by the physiologic effects of pregnancy (4). In the United States, in the last 3 decades, the ratio has more than doubled, rising from 7.2 deaths per 100,000 births in 1987 to 17.3 deaths per 100,000 births in 2013 (5). Similar to the developed countries, pregnancy-related mortality of the mother is high in China with a rate of 18.3 per 100,000, amongst which cardiovascular diseases account for the second highest percentage (15.7%) (6).

Significant QTc prolongation reflecting lengthened cardiac repolarization is associated with an increased risk of Torsade de Pointes tachycardia (TdP), ventricular fibrillation (VF), sudden cardiac death (SCD) and all-cause mortality in a general population (7). Studies reported that there were only modest prolongations in the QTc interval during pregnancy, which usually remained within the normal range (8, 9). We have observed that the QTc intervals are longer in twin pregnancies than in single pregnancy and non-pregnant women. Nevertheless, there are limited data on the relationship between pregnancy and QTc interval. In this study, we therefore aim to assess the prevalence of QTc prolongation in Chinese hospitalized parturient women with single and twin pregnancies, and to search for potential risk factors associated with QTc prolongation.

## Methods

### Study Population

This study is a retrospective analysis of 1,218 consecutive Chinese hospitalized parturient women with single and twin pregnancies, who were admitted to the First Affiliated Hospital of Xi'an Jiaotong University. We used random sampling method to draw 609 cases from the group of single pregnancy between January 2014 and October 2020 and analyzed them in comparison with the group of twin pregnancies in the same period. The inclusion criteria were: (1) previously full-term parturient women with single or twin pregnancies confirmed by prenatal ultrasonography; (2) candidates had at least one 12-lead surface ECG in sinus rhythm, which is routinely ordered for every hospitalized pregnant woman in our hospital; and (3) candidates received ECG measurements and laboratory tests within 72 h before delivery. Exclusion criteria were: (1) history or family history of congenital cardiac diseases including congenital LQTS; (2) ECG with other cardiac disorders (i.e., atrial flutter, atrial fibrillation, atrioventricular block and bundle branch block); and (3) structural heart disease. Informed consent was obtained from each patient and the study protocol conforms to the ethical guidelines of the 1975 Declaration of Helsinki as reflected in a prior approval by the First Affiliated Hospital of Xi'an Jiaotong University's human research committee (XJTU1AF2019LSK-056).

### ECG Evaluation

Twelve-lead surface ECGs were recorded at a paper speed of 25 mm/s and a voltage of 10 mm/ mV in the supine position. ECG parameters encompassed heart rate, QT, QTc, QRS, R_V5_ + S_V1_. All ECGs were processed in a central laboratory (Department of Cardiology, The First Affiliated Hospital of Xi'an Jiaotong University, Xi'an, Shaanxi, China), where they were visually inspected for technical errors and inadequate quality. ECGs were processed with the 2001 version of the Marquette 12-SL program (GE Marquette). The QT intervals were measured in all 12 leads, and the longest QT interval was taken for analysis. We defined as isoelectric the line drawn between the PR interval of one beat and the TP interval immediately after the corresponding T wave. The end of the T wave was defined as the intersection between the largest tangent of the terminal phase of the T wave and the isoelectric line. The T wave peak was measured at the greatest vertical amplitude of the T wave relative to the isoelectric line (10). QTc was corrected by the Fridericia formula [QTc = QT/RR(1/3)]. All tracings were studied by two independent investigators, and a consensus was reached in cases where there was a disagreement. QTc ≥ 460 ms for females was defined as prolonged QTc, and QTc ≥ 500 ms was defined as severely prolonged QTc (11).

### Clinical Data Collection

Basic information of parturient women with single and twin pregnancies were collected including age, systolic blood pressure (SBP), diastolic blood pressure (DBP), hemoglobin (Hb), liver function test (total bile acid, TBA), renal function test (blood glucose, GLU; serum uric acid, UA), blood lipid (total cholesterol, TC), serum electrolytes (serum potassium, K^+^; serum magnesium, Mg^2+^; serum calcium, Ca^2+^), gestational weight gain (GWG), fetal weight, and medical history (anemia; gestational hypertension; eclampsia; gestational diabetes mellitus, GDM; peripartum cardiomyopathy, PPCM; infection; first/second pregnancy) from the electronic medical records. All laboratory tests and ECG measurements were conducted within 72 h before delivery.

### Statistical Analysis

IBM SPSS Statistics V.22.0 (IBM Chicago, New York, USA) was employed for data analysis. Continuous variables were analyzed via Student's *t*-test and presented as mean ± SD. The Mann-Whitney *U*-test was applied for non-normally distributed variables. The box and whiskers plot exhibited the median, 25th and 75th percentiles and the range of QTc for single and twin pregnancies. Numbers and proportions (%) were used for categorical variables and were analyzed by the χ^2^ test or the Fisher's exact test. Less than 5% missing and incomplete data were handled by univariate imputation with median. Logistic regression model was used to compare single to twin pregnancies, with QTc as the dependent variable and the following variables as covariates: heart rate, SBP, DBP, hypertension, second pregnancy, TBA, GLU, TC, K^+^, Mg^2+^, Ca^2+^, UA, fetal weight, GWG, R_V5_+S_V1_. These variables were chosen because of the statistically significant difference in baseline characteristics between single and twin pregnancies. Uni-and multivariable logistic regression analyses were also applied to identify clinical parameters related to QTc prolongation. The results were considered significant when *p* < 0.05. The odds ratio (OR) and 95% confidence intervals (CI) were used to express the relationship between the QTc interval and the risk factors.

## Results

**Figure 1:**
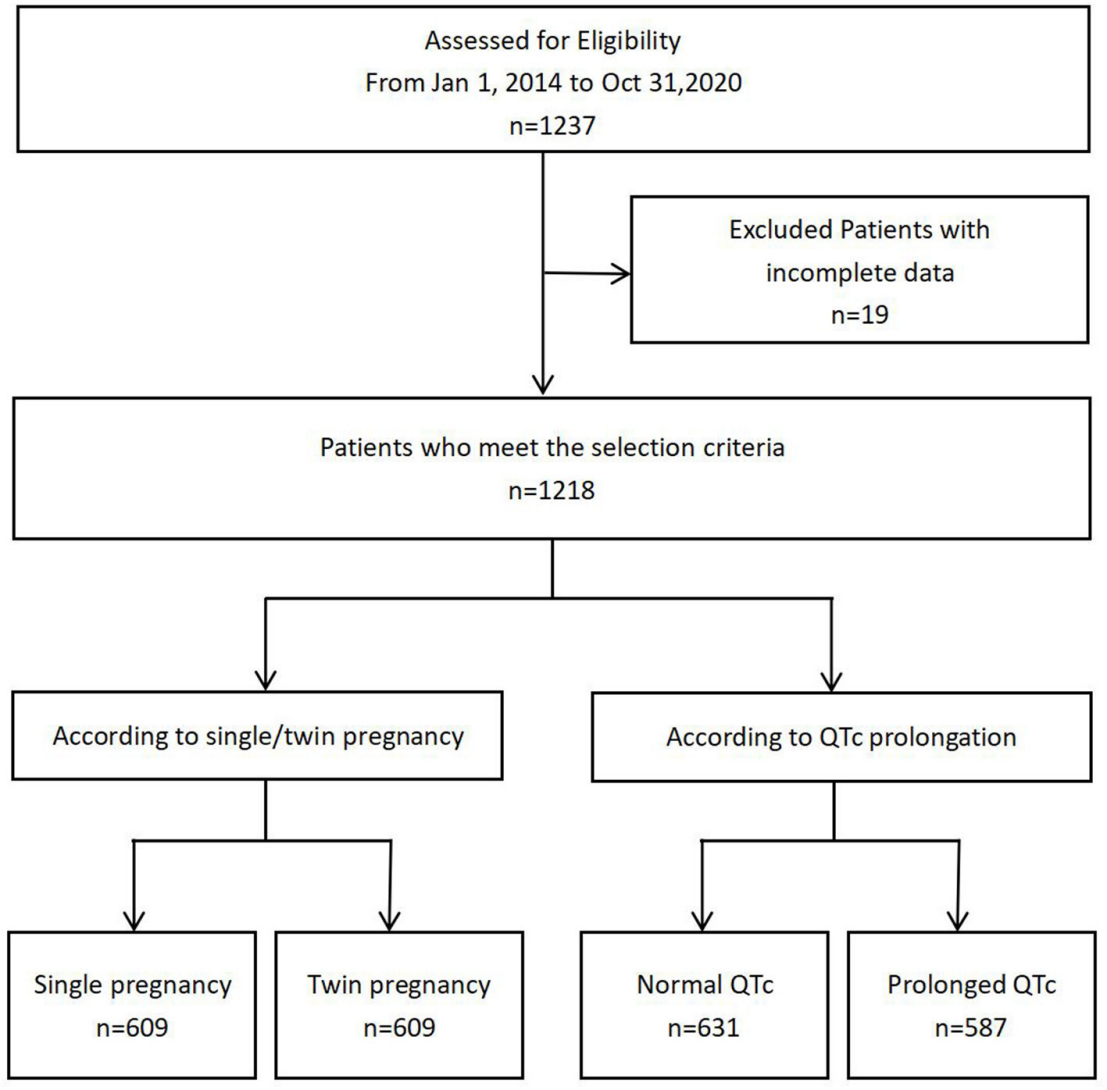
Flow diagram demonstrating patient inclusion and study workflow.

**Figure 2:**
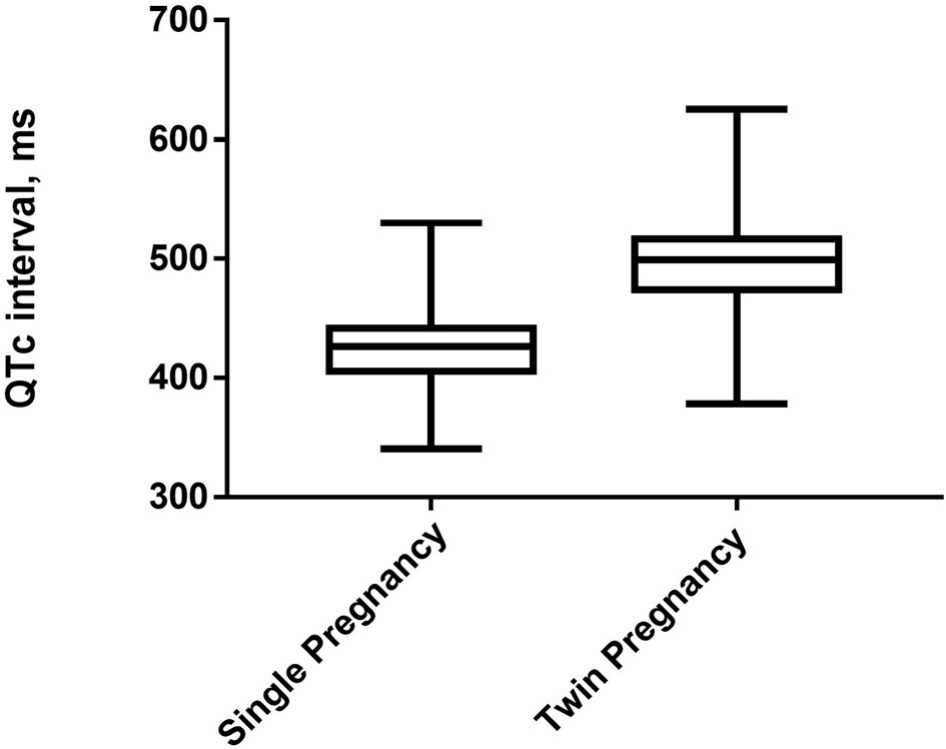
The box and whiskers plot exhibit the median, 25th and 75th percentiles and the range of QTc for single and twin pregnancies.

A total of 1,218 parturient women with single and twin pregnancies were included in this study (Figure 1). The candidates were randomly selected. Six hundred and nine participants had single pregnancy (50.00%), whereas 609 participants had twin pregnancies (50.00%). Their mean age was 30.48 ± 4.36 years old. The prevalence of QTc prolongation was 48.19% among the total cohort, 10.18% in single pregnancy and 86.21% in twin pregnancies. The proportion of severely prolonged QTc was 21.84% among the total cohort, 0.49% in single pregnancy, and 43.18% in twin pregnancies. The mean QTc interval was significantly longer in twin pregnancies than in single pregnancy (Figure 2).

### Comparison of Risk Factors Between Single and Twin Pregnancies

Several risk factors were identified to be significant different between single and twin pregnancies including SBP, DBP, TBA, GLU, TC, K^+^, Mg^2+^, Ca^2+^, UA, fetal weight, GWG, HR, QT, QTc, proportions of QTc prolongation, proportions of severe QTc prolongation, QRS, R_V5_+S_V1_, proportions of gestational hypertension and of second pregnancy in univariate analysis. Among those, multivariate analysis showed QTc prolongation still remained significantly different in single vs. twin pregnancies (Tables 1, 2).

**Table 1:** Compared of demographic and clinical characteristics between single and twin pregnancies.

**Index**	**Single (*n* = 609)**	**Twin (*n* = 609)**	***P*-value**
Age	30.94 ± 4.14	30.11 ± 4.09	0.087
SBP (mmHg)	120.86 ± 12.37	126.15 ± 15.93	<0.001
DBP (mmHg)	79.18 ± 10.16	82.12 ± 9.81	<0.001
Hb (g/L)	101.61 ± 14.46	100.85 ± 19.47	0.439
TBA (μmol/L)	3.64 ± 5.63	5.06 ± 5.98	<0.001
GLU (mmol/L)	4.97 ± 1.02	4.78 ± 1.31	0.005
TC (mmol/L)	5.78 ± 1.13	6.16 ± 1.42	<0.001
K^+^ (mmol/L)	3.87 ± 0.32	3.94 ± 0.36	<0.001
Mg^2+^ (mmol/L)	0.92 ± 0.09	0.93 ± 0.17	0.018
Ca^2+^ (mmol/L)	2.25 ± 0.16	2.22 ± 0.17	0.005
UA (μmol/L)	310.46 ± 70.88	354.96 ± 101.69	<0.001
hsCRP (mg/L)	1.98 ± 0.42	2.14 ± 0.23	0.067
Fetal weight (g)	3,228.19 ± 481.88	4,743.57 ± 938.81	<0.001
GWG (kg)	15.25 ± 4.40	18.45 ± 6.38	<0.001
HR (bpm)	85.38 ± 12.33	76.68 ± 15.41	<0.001
QT (ms)	380.00 ± 30.78	463.91 ± 48.42	<0.001
QTc (ms)	424.96 ± 27.67	498.65 ± 38.24	<0.001
QTc prolongation (%)	62 (10.18%)	525 (86.21%)	<0.001
Severe QTc prolongation (%)	3 (0.49%)	263 (43.18%)	<0.001
QRS (ms)	97.50 ± 14.92	108.44 ± 20.91	0.008
R_V5_ + S_V1_ (mv)	1.62 ± 0.46	1.94 ± 0.62	<0.001
Anemia (%)	442 (72.58%)	437 (71.76%)	0.749
Hypertension (%)	53 (8.70%)	140 (22.99%)	<0.001
Eclampsia (%)	34 (5.58%)	65 (10.67%)	0.057
GDM (%)	52 (8.54%)	40 (6.57%)	0.658
PPCM (%)	6 (0.98%)	19 (3.12%)	0.432
Infection (%)	51 (8.37%)	29 (4.76%)	0.074
First/second pregnancy			0.033
First (%)	365 (59.93%)	401 (65.85%)	
Second (%)	244 (40.07%)	208 (34.15%)	

*SBP, systolic blood pressure; DBP, diastolic blood pressure; Hb, hemoglobin; TBA, total bile acid; GLU, blood glucose; TC, total cholesterol; K^+^, serum potassium; Mg^2+^, serum magnesium; Ca^2+^, serum calcium; UA, serum uric acid; GWG, gestation weight gain; HR, heart rate; QTc, corrected QT; GDM, gestational diabetes mellitus; PPCM, peripartum cardiomyopathy*.

**Table 2:** Association between QTc prolongation and single vs. twin pregnancies.

**Characteristics**	**OR (95%CI)**	***P* value**
**QTc prolongation** (**QT** > **460 ms**)
Model 1	1.246 (1.126–1.563)	<0.001
Model 2	1.214 (1.109–1.480)	<0.001
Model 3	1.187 (1.102–1.316)	<0.001
Model 4	1.142 (1.097–1.223)	<0.001
**Severe QTc prolongation** (**QT** > **500 ms**)
Model 1	1.427 (1.060–1.921)	<0.001
Model 2	1.395 (1.142–1.705)	<0.001
Model 3	1.286 (1.094–1.501)	<0.001
Model 4	1.197 (1.032–1.368)	<0.001

*Model 1 QTc prolongation or severe QTc prolongation*.

*Model 2 adjusted for model 1 plus HR*.

*Model 3 adjusted for model 2 plus SBP, DBP, Hypertension, Second pregnancy, Infection*.

*Model 4 adjusted for model 3 plus TBA, GLU, TC,K^+^, Mg^2+^, Ca^2+^, UA, hsCRP, Fetal weight, GWG, R_V5_ + S_V1_*.

### Comparison of Risk Factors Between Normal QTc and Prolonged QTc Groups

Differences in the following risk factors have been revealed to be statistically significant between the prolonged QTc and the normal QTc group: SBP, DBP, TC, UA, fetal weight, QRS duration, R_V5_ + S_V1_. The incidence of gestational hypertension, eclampsia, PPCM and twin pregnancies were significantly higher in the prolonged QTc as compared to the normal QTc group (Table 3). In univariable logistic regression analysis, age, SBP, DBP, GLU, TC, K^+^, Mg^2+^, Ca^2+^, UA, fetal weight, GWG, proportion of hypertension, eclampsia, PPCM and twin pregnancies showed a correction with QTc prolongation. In multivariable logistic regression analysis, SBP (OR 1.058, 95%CI 1.035–1.081), DBP (OR 1.054, 95%CI 1.016–1.082), TC (OR 1.473, 95%CI 1.231–1.726) and serum UA (OR 1.033, 95%CI 1.012–1.058) were significantly associated with QTc prolongation in parturient women. Compared to candidates without obstetric complications, gestational hypertension (OR 1.838, 95%CI 1.543–2.139) was associated with QTc prolongation. Parturient women with twin pregnancies (OR 7.122, 95%CI 6.143–9.912) were more prone to exhibit QTc prolongation (Table 4). The ROC curve of multivariable logistic regression was also delineated (Supplementary Figure 1). The area under ROC curve (AUC) was 0.917, the prediction accuracy of this model was 85.4%.

**Table 3:** Compared of demographic and clinical characteristics between QTc-normal and QTc-prolongation groups.

**Index**	**Normal QTc**	**Prolonged QTc**	***P*-value**
	**(*n* = 631)**	**(*n* = 587)**	
Age	30.66 ± 4.19	30.38 ± 4.08	0.567
SBP (mmHg)	121.28 ± 13.17	125.86 ± 15.48	0.028
DBP (mmHg)	79.01 ± 9.88	82.40 ± 10.03	0.009
Hb (g/L)	101.87 ± 14.82	100.55 ± 19.32	0.213
TBA (μmol/L)	4.20 ± 6.26	4.52 ± 5.38	0.526
GLU (mmol/L)	4.91 ± 1.06	4.84 ± 1.30	0.287
TC (mmol/L)	5.78 ± 1.23	6.18 ± 1.34	0.001
K^+^ (mmol/L)	3.88 ± 0.34	3.94 ± 0.34	0.547
Mg^2+^ (mmol/L)	0.91 ± 0.10	0.94 ± 0.17	0.976
Ca^2+^ (mmol/L)	2.25 ± 0.16	2.22 ± 0.17	0.255
UA (μmol/L)	309.61 ± 75.24	357.55 ± 98.45	0.005
hsCRP (mg/L)	1.96 ± 0.65	2.09 ± 0.71	0.113
Fetal weight (g)	3,402.61 ± 686.74	4,612.87 ± 1,039.95	<0.001
GWG (kg)	15.56 ± 4.70	18.23 ± 6.33	0.308
HR (bpm)	84.71 ± 14.10	77.08 ± 14.11	0.684
QT (ms)	378.92 ± 29.77	468.21 ± 44.54	<0.001
QTc (ms)	422.09 ± 23.12	504.50 ± 32.15	<0.001
QRS (ms)	95.47 ± 15.05	111.04 ± 19.43	<0.001
R_V5_ + S_V1_ (mv)	1.67 ± 0.48	1.90 ± 0.63	0.038
Anemia (%)	451 (71.47%)	428 (72.91%)	0.575
Hypertension (%)	57 (9.03%)	136 (23.17%)	<0.001
Eclampsia (%)	41 (6.50%)	58 (9.88%)	0.031
GDM (%)	50 (7.92%)	42 (7.15%)	0.612
PPCM (%)	6 (0.95%)	19 (3.24%)	0.005
Infection (%)	38 (6.02%)	42 (7.15%)	0.425
Single/twin pregnancies			<0.001
Single (%)	547 (86.69%)	62 (10.56%)	
Twin (%)	84 (13.31%)	525 (89.44%)	
First/second pregnancy			0.649
First (%)	393 (62.28%)	373 (63.54%)	
Second (%)	238 (37.72%)	214 (36.46%)	

*Normal QTc < 460 ms; prolonged QTc ≥ 460 ms*.

*SBP, systolic blood pressure; DBP, diastolic blood pressure; Hb, hemoglobin; TBA, total bile acid; GLU, blood glucose; TC, total cholesterol; K^+^, serum potassium; Mg^2+^, serum magnesium; Ca^2+^, serum calcium; UA, serum uric acid; hsCRP, GWG, gestation weight gain; HR, heart rate; QTc, corrected QT; GDM, gestational diabetes mellitus; PPCM, peripartum cardiomyopathy*.

**Table 4:** Multivariable logistic regression analysis indicated risk factors significantly correlated with QTc prolongation in parturient.

**Variables**	**Multivariable**	
	**OR (95%CI)**	***P*-value**
Age	0.962 (0.917–1.081)	0.156
SBP (mmHg)	1.058 (1.035–1.081)	<0.001
DBP (mmHg)	1.054 (1.016–1.082)	<0.001
TC (mmol/L)	1.473 (1.231–1.726)	<0.001
UA (μmol/L)	1.033 (1.012–1.058)	<0.001
Fetal weight (g)	1.002 (1.001–1.003)	0.351
HR (bpm)	0.981 (0.927–1.038)	0.513
Hypertension (%)	1.838 (1.543–2.139)	0.001
Eclampsia (%)	1.153 (0.921–1.479)	0.198
PPCM (%)	2.337 (0.428–7.653)	0.385
Twin pregnancy (%)	7.122 (6.143–9.912)	<0.001

*SBP, systolic blood pressure; DBP, diastolic blood pressure; TC, total cholesterol; UA, serum uric acid; HR, heart rate; PPCM, peripartum cardiomyopathy*.

### Comparison of Risk Factors Between QTc < 500 ms and QTc ≥ 500 ms Groups

Differences in the following risk factors have been revealed to be statistically significant between the QTc < 500 ms and QTc ≥ 500 ms groups: SBP, DBP, UA, fetal weight, QT, QTc, QRS duration. The incidence of gestational hypertension, eclampsia, PPCM and twin pregnancies were significantly higher in the QTc ≥ 500 ms groups as compared to the QTc < 500 ms group (Table 5). In multivariable logistic regression analysis, SBP (OR 1.539, 95%CI 1.035–2.218), DBP (OR 1.505, 95%CI 1.162–2.261), serum UA (OR 1.257, 95%CI 1.027–1.538) were significantly associated with severe QTc prolongation in parturient women. Compared to candidates without obstetric complications, gestational hypertension (OR 1.894, 95%CI 1.613–2.242), was associated with severe QTc prolongation. Parturient women with twin pregnancies (OR 11.776, 95%CI 5.269–21.672) were more prone to exhibit severe QTc prolongation (Table 6).

**Table 5:** Compared of demographic and clinical characteristics between QTc < 500 ms and QTc ≥ 500 ms groups.

**Index**	**QTc <500 ms**	**QTc ≥500 ms**	***P*-value**
	**(*n* = 952)**	**(*n* = 266)**	
Age	29.94 ± 5.30	30.38 ± 3.01	0.245
SBP (mmHg)	120.28 ± 14.76	124.17 ± 12.59	0.017
DBP (mmHg)	80.12 ± 10.56	82.94 ± 7.32	0.001
Hb (g/L)	100.87 ± 16.24	100.55 ± 14.59	0.237
TBA (μmol/L)	4.72 ± 5.12	4.21 ± 2.17	0.715
GLU (mmol/L)	4.78 ± 1.94	4.38 ± 1.01	0.324
TC (mmol/L)	5.50 ± 2.16	5.74 ± 0.95	0.611
K^+^ (mmol/L)	3.76 ± 0.54	3.85 ± 0.21	0.874
Mg^2+^ (mmol/L)	0.91 ± 0.14	0.93 ± 0.08	0.943
Ca^2+^ (mmol/L)	2.24 ± 0.22	2.22 ± 0.10	0.726
UA (μmol/L)	312.18 ± 95.90	347.64 ± 68.33	0.007
hsCRP (mg/L)	2.21 ± 0.87	2.01 ± 0.51	0.372
Fetal weight (g)	3,528.12 ± 736.45	4,652.33 ± 874.24	<0.001
GWG (kg)	16.40 ± 6.03	17.38 ± 4.33	0.248
HR (bpm)	82.92 ± 17.47	78.24 ± 12.15	0.659
QT (ms)	419.92 ± 48.30	527.18 ± 15.76	<0.001
QTc (ms)	440.17 ± 32.50	527.04 ± 16.24	<0.001
QRS (ms)	90.05 ± 17.28	144.88 ± 6.95	<0.001
R_V5_ + S_V1_ (mv)	1.70 ± 0.72	1.83 ± 0.51	0.244
Anemia (%)	684 (71.84%)	195 (73.30%)	0.575
Hypertension (%)	58 (6.09%)	135 (50.75%)	<0.001
Eclampsia (%)	28 (2.94%)	71 (26.70%)	<0.001
GDM (%)	72 (7.56%)	20 (7.52%)	0.887
PPCM (%)	9 (0.94%)	16 (6.02%)	0.001
Infection (%)	63 (6.62%)	17 (6.39%)	0.612
Single/twin pregnancies			<0.001
Single (%)	606 (63.64%)	3 (1.13%)	
Twin (%)	346 (36.34%)	263 (98.87%)	
First/second pregnancy			0.546
First (%)	599 (62.92%)	167 (62.78%)	
Second (%)	353 (37.08%)	99 (37.22%)	

**Table 6:** Multivariable logistic regression analysis indicated risk factors significantly correlated with severe QTc prolongation in parturient women.

	**Multivariable**	
	**OR (95%CI)**	***P*-value**
**Variables**
Age	1.029 (0.947–1.108)	0.231
SBP (mmHg)	1.539 (1.035–2.218)	0.033
DBP (mmHg)	1.505 (1.162–2.261)	0.007
UA (μmol/L)	1.257 (1.027–1.538)	0.026
Fetal weight (g)	1.345 (0.623–2.905)	0.461
HR (bpm)	0.922 (0.471–2.132)	0.995
Hypertension (%)	1.894 (1.613–2.242)	0.001
Eclampsia (%)	1.299 (0.861–1.589)	0.572
PPCM (%)	2.687 (0.707–4.230)	0.810
Twin pregnancy (%)	11.776 (5.269–21.672)	<0.001

*SBP, systolic blood pressure; DBP, diastolic blood pressure; UA, serum uric acid; HR, heart rate; PPCM, peripartum cardiomyopathy*.

### Risk Factors of QTc Prolongation in Single Pregnancy

The prevalence of prolonged QTc was 10.18% in single pregnancy. Several clinical parameters were statistically different between the QTc-prolongation group and the QTc-normal group in single pregnancy, DBP, TC, serum Ca^2+^, hsCRP, heart rate. The incidence of gestational hypertension, infection and parity showed significant differences between the two QTc groups (Supplementary Table 1). In multivariable logistic regression analysis (Supplementary Table 2), DBP (OR 1.284, 95%CI 1.175–1.317), TC (OR 1.415, 95%CI 1.107–1.833) and hsCRP (OR 1.014, 95%CI 1.007–1.201) were associated with QTc prolongation in single pregnancy. The high incidence of gestational hypertension (OR 14.863, 95%CI 4.937–68.766), infection (OR 7.921, 95%CI 4.012–15.438) and second pregnancy (OR 2.941, 95%CI 1.366–4.648) were also associated with prolonged QTc in single pregnancy. The ROC curve of multivariable logistic regression was also delineated (Supplementary Figure 2). The AUC was 0.746, the prediction accuracy of this model was 87.2%.

### Risk Factors of QTc Prolongation in Twin Pregnancies

The prevalence of prolonged QTc was 86.21% in twin pregnancies. Factors including age, TBA, serum Mg^2+^, UA, hsCRP, fatal weight, HR, and incident of infection were significantly different between the QTc-prolongation and QTc-normal groups in twin pregnancies (Supplementary Table 3). In multivariable logistic regression analysis (Supplementary Table 4), increased age (OR 1.213, 95%CI 1.116–1.307), TBA (OR 0.946, 95%CI 0.892–0.985), UA (OR 1.037, 95%CI 1.018–1.049), hsCRP (OR 1.089, 95%CI 1.022–1.153) and incident of infection (OR 4.539, 95%CI 1.493–9.125) were associated with QTc prolongation in twin pregnancies. The ROC curve of multivariable logistic regression was delineated (Supplementary Figure 3). The AUC was 0.726, the prediction accuracy of this model was 84.3%.

## Discussion

In this retrospective study, we analyzed a total of 1,218 parturient women with single or twin pregnancies to assess the prevalence and risk factors of QTc prolongation. Firstly, the prevalence of QTc prolongation and severely prolonged QTc were much higher in twin pregnancies as compared to single pregnancy. This novel finding suggests that parturient women with twin pregnancies are more susceptible to QTc prolongation, which is a risk factor for potentially lethal ventricular arrhythmias such as TdP, VF and SCD. Secondly, systemic arterial blood pressure (SBP and DBP), TBA, TC, UA, fetal weight, GWG, QT, QTc, proportions of QTc prolongation, proportions of severe QTc prolongation, QRS, R_V5_ + S_V1_, and the incidence of gestational hypertension were significantly higher in twin pregnancies than in single pregnancy. Among these factors, mean QT intervals remained within the normal range in single pregnancy, but slightly prolonged in the twin pregnancies. Candidates with single pregnancy were in the upper limit of normal QTc intervals, which is consistent with previous studies that have shown only modest prolongations in the QTc interval during pregnancy, which usually remain within the normal range. However, QTc intervals in our study were longer in twin pregnancies. These results indicate that pregnant women with twin pregnancies carry a higher risk of ventricular arrhythmias than single pregnancy. This is significant because QTc prolongation and its associated risk of TdP are known to be exacerbated by the presence of at least one risk factor (12), and it is especially important for pregnant women. It is also notable that the higher proportion of QTc prolongation occurred in twin pregnancies, indicating that a risk of repolarisation delay can occur in younger individuals possessing additional risk factors.

Our main finding is that QTc-prolongation is much more prevalent in twin pregnancies than in single pregnancy. A majority of pregnant women with twin pregnancies had QRS prolongation and severely prolonged QTc intervals, which enhance the heterogeneity of ventricular repolarization and predispose them to a higher risk of ventricular arrhythmias (13, 14). Myocardial hypertrophy has been reported as an adaptive change during pregnancy to support the increased metabolic demands of maternal consumption and fetal development (15, 16). In our study, values of R_V5_ + S_V1_ as a surrogate parameter of LV hypertrophy were higher in twin pregnancies than in single pregnancy, and the values of R_V5_ + S_V1_ were also significantly higher in the QTc-prolongation group, which is in concordance with previous studies showing that patients with hypertrophic cardiomyopathy often have QTc prolongation (17, 18). Accordingly, cardiac hypertrophy is an underlying risk factor for QTc prolongation during pregnancy.

Both human and animal studies suggest that there is fundamental remodeling of ion channels during pregnancy causing delayed ventricular repolarization, and an increase in the heterogeneity of the timing of repolarization throughout the ventricles (19, 20). The QTc interval is a visualized reflection of ventricular repolarization. The extended action potential duration (APD) leads to the prolonged QT interval, which is caused by an increased net inward current (i.e., increase of the I_Na−L_ or I_Ca_, or decrease of the I_Ks_ and I_Kr_). An enhanced net inward current due to either acquired conditions or inherited gene mutations causes intracellular Ca^2+^ loading, the occurrence of early and delayed afterdepolarization. These electrophysiological changes promote the spatial and temporal dispersion of ventricular repolarization and finally lead to reentrant arrhythmias causing TdP, VF and SCD (21, 22). The behaviors of ion channels are complicated by multiple mechanisms including alterations in heteromeric combinations, ancillary subunit expression, intracellular messengers, changes in pH, hormonal status, and ischemic conditions during pregnancy. Despite unclear underlying mechanisms of QTc prolongation, there exist changes in the susceptibility to arrhythmias, and changes in the ability of drugs to prolong QTc during pregnancy (23). The physiological changes of pregnancy may alter drug properties affecting both mother and fetus. Therefore, medication during pregnancy should be carefully assessed in case of drug-induced arrhythmias (24).

The obstetric complications were also found to exacerbate QTc prolongation. Hypokalemia is strong independent risk factors for QTc prolongation (25). Anemia is a frequent complication in pregnancy. The mechanism of anemia causing QTc prolongation might due to reduced myocardial oxygen supply and autonomic dysfunction (26). The increased TC level was shown to be related to QTc prolongation in our study. Studies demonstrate that progesterone can impair hERG channel by disrupting intracellular cholesterol homeostasis. Exogenous application of cholesterol mimics the prohibitive effect of progesterone on hERG channel trafficking to cause QTc prolongation during late pregnancy, which support our findings (27). Hyperuricemia is a risk factor for coronary heart disease, but no report indicates the relationship between the increased serum UA and QTc prolongation (28, 29). The level of serum UA was higher in QTc-prolongation group with significant difference in our study, which provide a new evidence that increased serum UA might be related to QTc prolongation. Hypertension can significantly reduce the inward currents and increase the outward current during the action potential plateau, lead to APD prolongation, which is consistent with our result (30, 31). Maternal obesity is associated with many obstetric complications, such as fetal overgrowth, fetal malformations, gestational diabetes, thromboembolic complications, and hypertensive complications (32).

Pregnancy is a dramatic and dynamic period during a woman's life with many physiological adaptations of all maternal organs. The cardiovascular system is notably adaptable to the physiological, hormonal and metabolic stimuli with acute and chronic alterations. Estrogen, progesterone, oxygen uptake, heart rate, stroke volume, cardiac output, SBP and DBP show an obvious escalating trend during pregnancy, particularly in the 3rd trimester (33–35). Estrogen could prolong the QT interval, but there have been inconsistent reports in the literature on whether progesterone prolongs the QT interval. Some literature indicated that progesterone shortened QT duration and the effects of estradiol and progesterone on QT were opposite (36, 37). Although the effect of progesterone on QT is still a matter of debate, a shortening effect seems to be predominantly reported. However, no cross-linked effect of progesterone and estrogen on the QT interval in pregnancy has been reported in the literature so far, and we can only determine that the QT interval is longer in pregnancy than in non-pregnant women. We speculate that the effect of estrogen on the QT interval may be greater than that of progesterone in pregnant women. Moreover, due to the influence of sex hormones on the QTc interval, differences in hormone pattern in single and twin pregnancies should be taken seriously. The plasma P concentration was significantly higher in twin pregnancies than in single pregnancy and the levels of E2 did not show so significant difference like P between single and twin pregnancies in any of the weeks studied (38). This strengthens our confirmation that the prolongation of the QT interval during pregnancy receives not only estrogen and progesterone, but may also be influenced by other hormones or metabolites, such as thyroid hormones, estriol, etc. In addition, since sympathetic responses are different in single vs. twin pregnancies, this may also account for the difference in QT interval between single and twin pregnancies, and the specific mechanism still needs to be further explored.

## Conclusions

To our knowledge, this is the first study to assess differences in QTc prolongation between single and twin pregnancies. The prevalence of QTc prolongation is higher in parturient women, particularly in twin pregnancies. Therefore, more research is needed to investigate the mechanisms underlying QTc prolongation during pregnancy. Prophylactic measures such as prolonged cardiac monitoring and avoidance of QTc prolonging drugs may be warranted in pregnancy, particularly in twin pregnancies.

## Limitations

There remain some limitations in our study. Firstly, despite investigating a large cohort, because of the limited number of twin pregnancies, some clinical parameters did not reach statistical significance. In addition, we opt for completely random sampling rather than matched sampling neglected control for confounding. Secondly, information of maternal echocardiography and sexual hormones were not obtained since these were not routine clinical practice in our hospital. Thirdly, levels of different thyroid hormones were not detected during hospitalization. Fourthly, in view of our sample size, we could not do sensitivity analyses exclusively focused on healthy women without discussed comorbidities now and we would like to expand the study population and study the differences in QTc in the healthy women in our next research. Fifthly, our study was a cross-sectional study and we did not record ECG baseline before pregnancy, future studies need to observe the changes in QT interval during pregnancy. At last, we did not observe any case of potentially lethal ventricular arrhythmia. Consequently, an even larger prospective registry should be designed to address this question.

## Data Availability Statement

The original contributions presented in the study are included in the article/Supplementary Material, further inquiries can be directed to the corresponding author/s.

## Ethics Statement

The studies involving human participants were reviewed and approved by The First Affiliated Hospital of Xi'an Jiaotong University's Human Research Committee (XJTU1AF2019LSK-056). The patients/participants provided their written informed consent to participate in this study.

## Author Contributions

CL, ZD, YJ, PL, YY, and DH designed the study. DH and CL analyzed and interpreted the data. CL, ZD, and DH drafted the manuscript. DH, ZD, and YY revised the manuscript for important intellectual content. All authors contributed to the article and approved the submitted version.

## Funding

This study was supported by the Natural Science Basic Research Program of Shaanxi Province, China (2021JQ-394).

## Conflict of Interest

The authors declare that the research was conducted in the absence of any commercial or financial relationships that could be construed as a potential conflict of interest.

## Publisher's Note

All claims expressed in this article are solely those of the authors and do not necessarily represent those of their affiliated organizations, or those of the publisher, the editors and the reviewers. Any product that may be evaluated in this article, or claim that may be made by its manufacturer, is not guaranteed or endorsed by the publisher.

## Supplementary Material

Supplementary Figure S1 Receiver operating characteristics (ROC) curve of multivariable logistic regression in total study population.

Supplementary Figure S2 Receiver operating characteristics (ROC) curve of multivariable logistic regression in single pregnancy.

Supplementary Figure S3 Receiver operating characteristics (ROC) curve of multivariable logistic regression in twin pregnancies.

Supplemental Table S1 Compared of demographic and clinical characteristics between normal QTc and prolonged QTc in single pregnancy.

Supplemental Table S2 Multivariable logistic regression indicated risk factors significantly correlated with QTc prolongation in single pregnancy.

Supplemental Table S3 Compared of demographic and clinical characteristics between normal QTc and prolonged QTc in twin pregnancies.

Supplemental Table S4 Multivariable logistic regression analysis indicated risk factors significantly correlated with QTc prolongation in twin pregnancy.
