# Supplementary figures and images for: Prevalence and Risk Factors of QTc Prolongation During Pregnancy
Source: Front Cardiovasc Med. 2022 Jan 24;8:819901. doi: 10.3389/fcvm.2021.819901 (PMC8818739; doi:10.3389/fcvm.2021.819901)

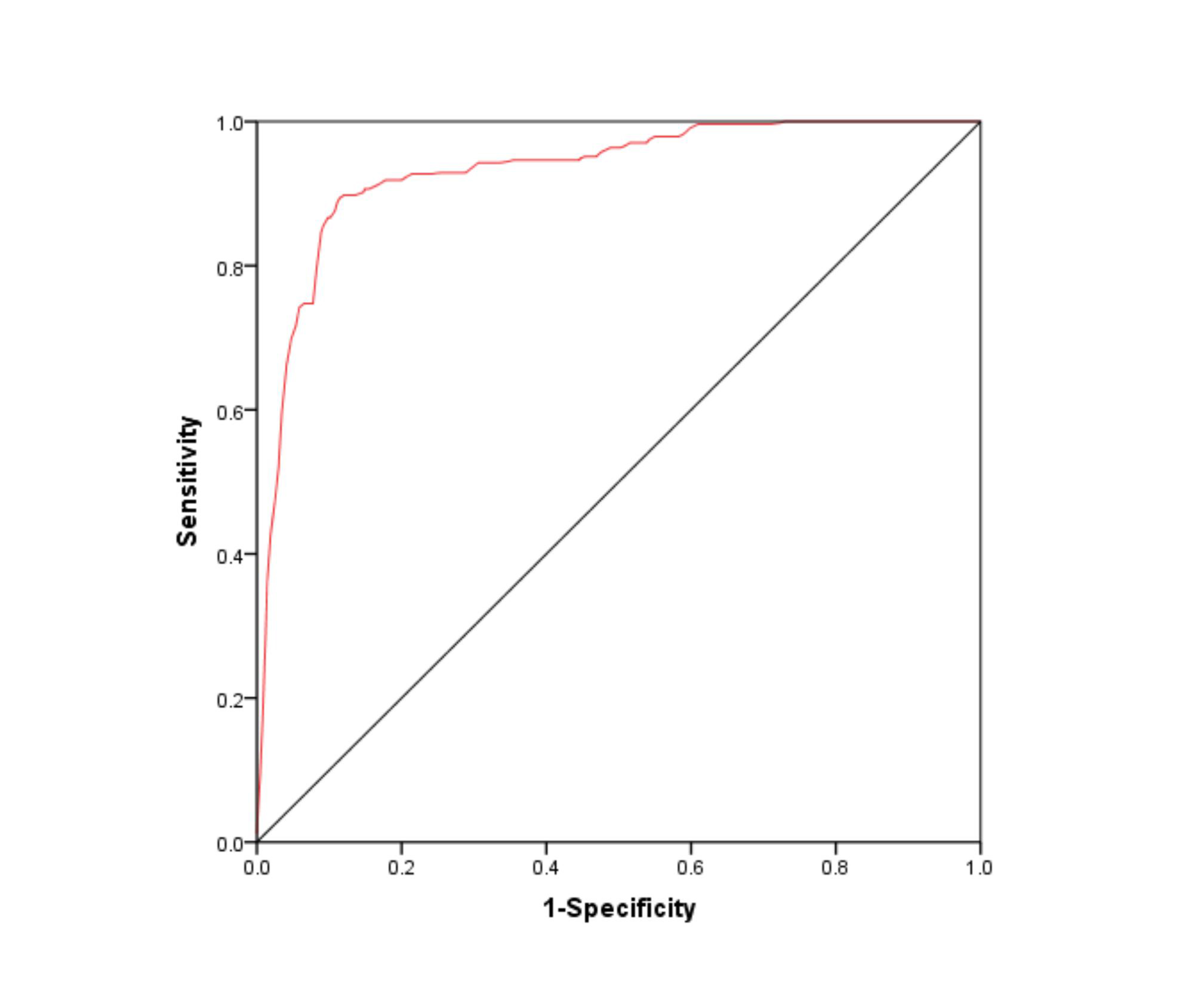

Supplement: Supplementary Figure S1 — Receiver operating characteristics (ROC) curve of multivariable logistic regression in total study population. [file Image_1.JPG]

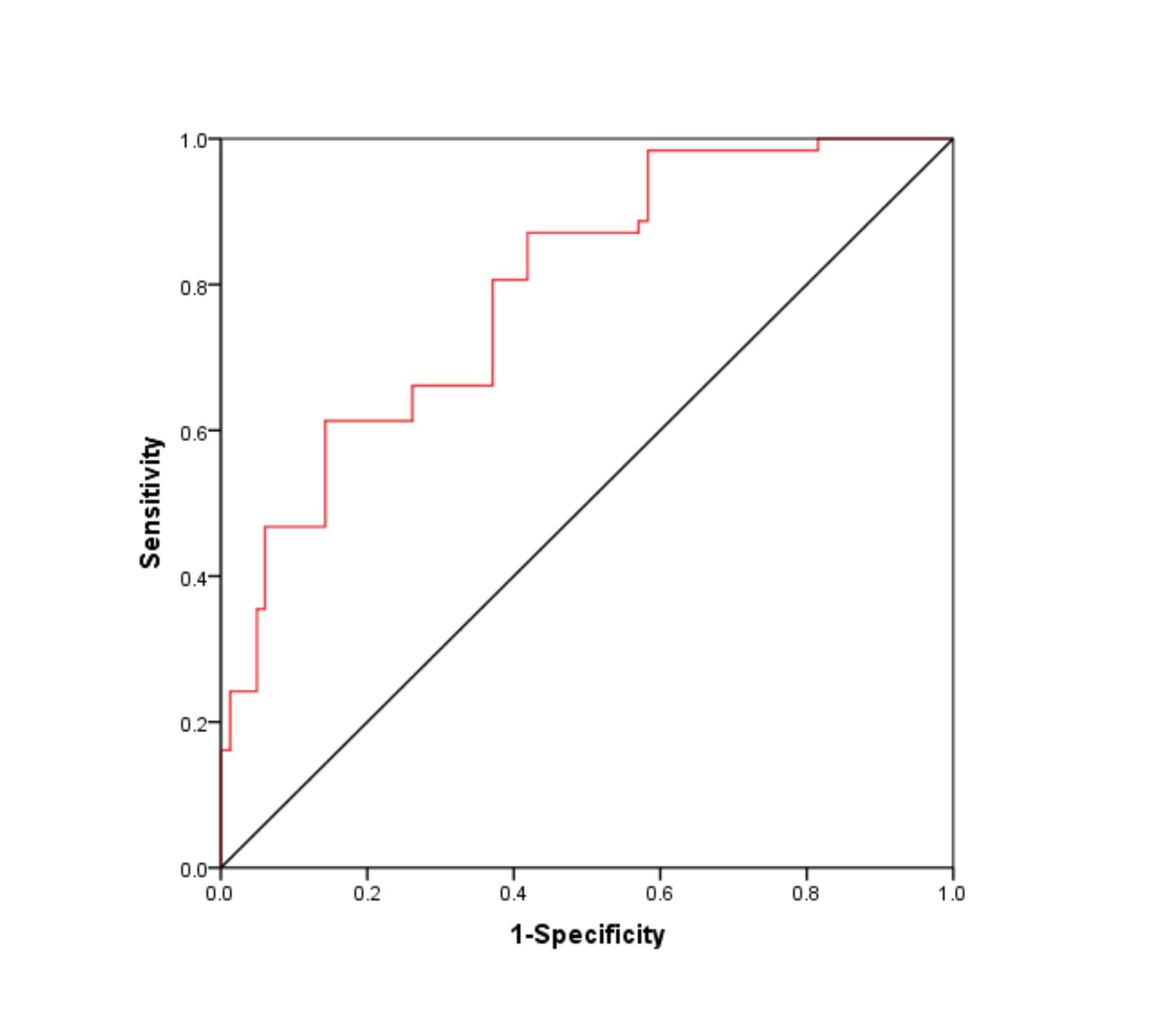

Supplement: Supplementary Figure S2 — Receiver operating characteristics (ROC) curve of multivariable logistic regression in single pregnancy. [file Image_2.JPG]

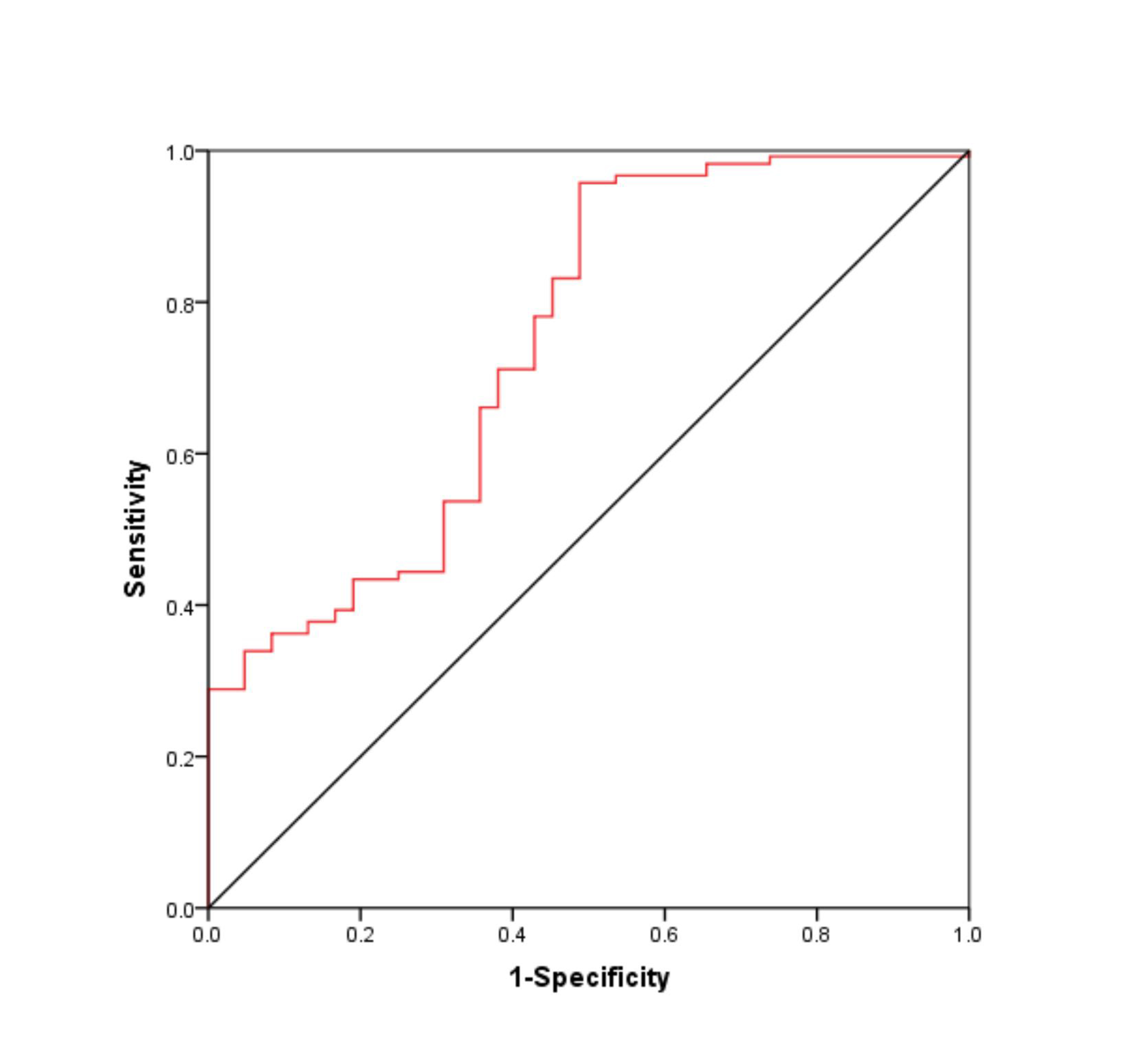

Supplement: Supplementary Figure S3 — Receiver operating characteristics (ROC) curve of multivariable logistic regression in twin pregnancies. [file Image_3.JPG]
