# Supplementary material for: Prevalence and Risk Factors of QTc Prolongation During Pregnancy
Source: Front Cardiovasc Med. 2022 Jan 24;8:819901. doi: 10.3389/fcvm.2021.819901 (PMC8818739; doi:10.3389/fcvm.2021.819901)
Supplement: Supplemental Table S1 — Compared of demographic and clinical characteristics between normal QTc and prolonged QTc in single pregnancy. [file Data_Sheet_1.zip › Table S1.DOCX]

| Index | Normal QTc  (n=547) | Prolonged QTc  (n=62) | P value |
| --- | --- | --- | --- |
| Age | 30.89±4.20 | 31.39±3.45 | 0.370 |
| SBP (mmHg) | 120.63±12.51 | 122.47±11.03 | 0.269 |
| DBP (mmHg) | 78.74±10.05 | 83.03±10.37 | 0.002 |
| Hb (g/L) | 101.86±14.57 | 99.45±13.34 | 0.214 |
| TBA (μmol/L) | 3.71±5.87 | 3.07±2.71 | 0.399 |
| GLU (mmol/L) | 5.18±1.12 | 5.01±1.33 | 0.373 |
| TC (mmol/L) | 4.96±1.04 | 5.08±0.84 | 0.157 |
| K^+^ (mmol/L) | 3.88±0.33 | 3.81±0.25 | 0.123 |
| Mg^2+^ (mmol/L) | 0.92±0.10 | 0.92±0.06 | 0.729 |
| Ca^2+^ (mmol/L) | 2.25±0.16 | 2.20±0.18 | 0.018 |
| UA (μmol/L) | 309.22±71.79 | 321.48±61.68 | 0.197 |
| hsCRP(mg/L) | 1.95±0.12 | 2.02±0.19 | 0.002 |
| Fetal weight (g) | 3238.81±445.95 | 3134.52±724.21 | 0.271 |
| GWG (kg) | 15.27±4.41 | 15.10±4.34 | 0.784 |
| HR (bpm) | 85.10±12.49 | 87.87±10.58 | 0.094 |
| QT (ms) | 375.47±27.97 | 420.00±25.09 | ＜0.001 |
| QTc (ms) | 419.33±22.52 | 474.68±17.12 | ＜0.001 |
| QRS (ms) | 96.16±14.75 | 109.35±10.69 | ＜0.001 |
| RV5+SV1 (mv) | 1.63±0.45 | 1.46±0.48 | 0.004 |
| Anemia (%) | 393(71.85%) | 49(79.03%) | 0.229 |
| Hypertension (%) | 43(7.86%) | 10(16.13%) | 0.029 |
| Eclampsia (%) | 29(5.30%) | 5(8.06%) | 0.369 |
| GDM (%) | 47(8.59%) | 5(8.06%) | 0.888 |
| PPCM (%) | 6(1.10%) | 0(0%) | 0.407 |
| Infection (%) | 38(6.95%) | 13(20.97%) | ＜0.001 |
| First/Second pregnancy | | | ＜0.001 |
| First (%) | 341(62.34%) | 24(38.71%) |  |
| Second (%) | 206(37.66%) | 38(61.29%) |  |

Supplemental Table 1: Compared of demographic and clinical characteristics between normal QTc and prolonged QTc in single pregnancy.

*

Normal QTc < 460 ms; prolonged QTc ≥ 460 ms.

Abbreviations: SBP, systolic blood pressure; DBP, diastolic blood pressure; Hb, hemoglobin; TBA, total bile acid; GLU, blood glucose; TC, total cholesterol; K^+^, serum potassium; Mg^2+^, serum magnesium; Ca^2+^, serum calcium; UA, serum uric acid; GWG, gestation weight gain; HR, heart rate; QTc, corrected QT; GDM, gestational diabetes mellitus; PPCM, peripartum cardiomyopathy
