# Supplementary material for: Prevalence and Risk Factors of QTc Prolongation During Pregnancy
Source: Front Cardiovasc Med. 2022 Jan 24;8:819901. doi: 10.3389/fcvm.2021.819901 (PMC8818739; doi:10.3389/fcvm.2021.819901)
Supplement: Supplemental Table S1 — Compared of demographic and clinical characteristics between normal QTc and prolonged QTc in single pregnancy. [file Data_Sheet_1.zip › Table S2.DOCX]

Supplemental Table 2: Multivariable logistic regression indicated risk factors significantly correlated with QTc prolongation in single pregnancy.

| Characteristics | OR (95%CI) | P value |
| --- | --- | --- |
| DBP | 1.284 (1.175-1.317) | <0.001 |
| TC | 1.415 (1.107-1.833) | 0.019 |
| Ca^+^ | 0.922 (0.873-1.082) | 0.184 |
| hsCRP(mg/L) | 1.014(1.007-1.201) | 0.044 |
| HR (bpm) | 1.108(0.968-1.231) | 0.793 |
| Hypertension | 14.863 (4.937-68.766) | 0.003 |
| Infection | 7.921 (4.012-15.438) | <0.001 |
| Second pregnancy | 2.941 (1.366-4.648) | 0.002 |

Abbreviations: DBP, diastolic blood pressure; TC, total cholesterol; Ca^2+^, serum calcium; HR, heart rate.
