# Supplementary material for: Prevalence and Risk Factors of QTc Prolongation During Pregnancy
Source: Front Cardiovasc Med. 2022 Jan 24;8:819901. doi: 10.3389/fcvm.2021.819901 (PMC8818739; doi:10.3389/fcvm.2021.819901)
Supplement: Supplemental Table S1 — Compared of demographic and clinical characteristics between normal QTc and prolonged QTc in single pregnancy. [file Data_Sheet_1.zip › Table S3.DOCX]

| Index | Normal QTc  (n=84) | Prolonged QTc (n=525) | P value |
| --- | --- | --- | --- |
| Age | 29.19±3.76 | 30.26±4.13 | 0.026 |
| SBP (mmHg) | 125.48±16.30 | 126.26±15.89 | 0.678 |
| DBP (mmHg) | 80.81±8.49 | 82.33±9.99 | 0.188 |
| Hb (g/L) | 101.98±16.45 | 100.67±19.92 | 0.570 |
| TBA (μmol/L) | 7.37±7.68 | 4.69±5.58 | ＜0.001 |
| GLU (mmol/L) | 4.61±1.13 | 4.81±1.35 | 0.194 |
| TC (mmol/L) | 5.88±1.62 | 6.21±1.39 | 0.079 |
| K^+^ (mmol/L) | 3.89±0.41 | 3.96±0.35 | 0.160 |
| Mg^2+^ (mmol/L) | 0.91±0.09 | 0.94±0.18 | 0.008 |
| Ca^2+^ (mmol/L) | 2.22±0.17 | 2.22±0.17 | 0.692 |
| UA (μmol/L) | 312.19±95.17 | 361.81±101.11 | <0.001 |
| hsCRP(mg/L) | 1.87±0.26 | 2.15±0.39 | 0.028 |
| Fetal weight (g) | 4469.29±971.18 | 4787.45±926.97 | 0.004 |
| GWG (kg) | 17.46±5.98 | 18.60±6.43 | 0.126 |
| HR (bpm) | 82.17±21.83 | 75.81±13.94 | 0.011 |
| QT (ms) | 401.43±31.48 | 473.90±42.87 | ＜0.001 |
| QTc (ms) | 440.06±18.55 | 508.02±31.69 | ＜0.001 |
| QRS (ms) | 90.95±16.25 | 111.24±20.21 | ＜0.001 |
| RV5+SV1 (mv) | 1.93±0.59 | 1.95±0.63 | 0.772 |
| Anemia (%) | 58(69.05%) | 379(72.19%) | 0.552 |
| Hypertension (%) | 14(16.67%) | 126(24.00%) | 0.138 |
| Eclampsia (%) | 12(14.29%) | 53(10.10%) | 0.248 |
| GDM (%) | 3(3.57%) | 37(7.05%) | 0.232 |
| PPCM (%) | 0(0.0%) | 19(3.62%) | 0.076 |
| Infection (%) | 0(0.0%) | 29(5.52%) | 0.027 |
| First/Second pregnancy | | | 0.412 |
| First (%) | 52(61.90%) | 349(57.31%) |  |
| Second (%) | 32(38.10%) | 176(33.52%) |  |

Supplemental Table 3: Compared of demographic and clinical characteristics between normal QTc and prolonged QTc in twin pregnancies.

Normal QTc < 460 ms; prolonged QTc ≥ 460 ms.

Abbreviations: SBP, systolic blood pressure; DBP, diastolic blood pressure; Hb, hemoglobin; TBA, total bile acid; GLU, blood glucose; TC, total cholesterol; K^+^, serum potassium; Mg^2+^, serum magnesium; Ca^2+^, serum calcium; UA, serum uric acid; GWG, gestation weight gain; HR, heart rate; QTc, corrected QT; GDM, gestational diabetes mellitus; PPCM, peripartum cardiomyopathy
