# Supplementary material for: Prevalence and Risk Factors of QTc Prolongation During Pregnancy
Source: Front Cardiovasc Med. 2022 Jan 24;8:819901. doi: 10.3389/fcvm.2021.819901 (PMC8818739; doi:10.3389/fcvm.2021.819901)
Supplement: Supplemental Table S1 — Compared of demographic and clinical characteristics between normal QTc and prolonged QTc in single pregnancy. [file Data_Sheet_1.zip › Table S4.DOCX]

Supplemental Table 4: Multivariable logistic regression analysis indicated risk factors significantly correlated with QTc prolongation in twin pregnancy.

| Characteristics | OR (95%CI) | P value |
| --- | --- | --- |
| Age | 1.213(1.116-1.307) | <0.001 |
| TBA (μmol/L) | 0.946(0.892-0.985) | 0.031 |
| Mg^2+^ (mmol/L) | 1.009(0.981-1.054) | 0.698 |
| UA (μmol/L) | 1.037(1.018-1.049) | 0.002 |
| hsCRP(mg/L) | 1.089(1.022-1.153) | 0.034 |
| Fetal weight (g) | 1.135(0.867-1.398) | 0.392 |
| HR (bpm) | 0.984(0.925-1.096) | 0.573 |
| Infection | 4.539(1.493-9.125) | 0.006 |

Abbreviations: TBA, total bile acid; Mg^2+^, serum magnesium; UA, serum uric acid; HR, heart rate.
